# Ethical issues in donation-based prosocial interventions for health: a scoping review

**DOI:** 10.1186/s12910-026-01559-4

**Published:** 2026-07-22

**Authors:** Takhona G. Hlatshwako, Delaney Santre, Suzanne Day, Dorian Ho, Weiming Tang, Jeremy Sugarman, Joseph D. Tucker

**Affiliations:** 1 Department of Health Policy and Management, University of North Carolina at Chapel Hill, Chapel Hill, NC USA; 2 Institute for Global Health and Infectious Diseases, University of North Carolina at Chapel Hill, 130 Mason Farm Rd, Chapel Hill, NC 27514 USA; 3 College of Arts and Sciences, University of North Carolina at Chapel Hill, Chapel Hill, NC USA; 4 School of Medicine, University of North Carolina at Chapel Hill, Chapel Hill, NC USA; 5 Icahn School of Medicine at Mt. Sinai, New York, NY USA; 6 Berman Institute of Bioethics and Department of Medicine, Johns Hopkins University, Maryland, USA

**Keywords:** Public Health Ethics, Organ Donation, Prosocial, Bioethics, Tissue Donation, Intervention

## Abstract

**Background:**

Donation-based prosocial interventions for health, such as monetary donations for diagnostic tests, aim to encourage voluntary actions that benefit others while fostering public participation to promote health. These interventions are increasingly used in public health, but the associated ethical issues have not been comprehensively described.

**Methods:**

We conducted a scoping review using the Arksey and O’Malley framework to synthesize bioethics and public health literature on the ethical issues encountered with donation-based prosocial interventions for health. We searched PubMed, PsycINFO, SCOPUS, NIH Reporter, and ClinicalTrials.gov for empirical qualitative, quantitative, and mixed-methods studies. Two independent reviewers extracted data and coded data from each study according to a codebook. Categories of similar codes were analyzed to name cross-cutting issues across the included studies.

**Results:**

The search identified 2,585 citations; 34 publications were included in our review. The publications described studies involving donations of blood, organs, biospecimens, data, and money. Most studies (32/34) were conducted in high-income countries and used observational designs (31/34). We identified three broad categories of benefits associated with donation-based interventions: health (physical and psychological), social (generosity, kindness, and community cohesion), and economic (improving welfare). We also identified several problematic ethical concerns at different levels (donor, recipient, provider, and system), such as undue pressure, privacy concerns, and inaccurate information. To mitigate these concerns, the literature discussed strengthening informed consent, ensuring confidentiality, building public trust, and ensuring appropriate stewardship of donations. We found that most studies did not empirically measure adverse consequences associated with donation-based prosocial interventions.

**Conclusions:**

Donation-based prosocial interventions offer several health, social, and economic benefits. However, it is essential to consider the potential ethical concerns that may arise in their design and dissemination. Further research is needed to evaluate the success of mitigation strategies for addressing these concerns and thereby enhance the overall benefits of prosocial interventions.

**Supplementary Information:**

The online version contains supplementary material available at https://doi.org/10.1186/s12910-026-01559-4.

## Background

Prosocial interventions (such as donations of time, money, blood, organs, data, or biospecimens) have become more common in public health [1]. Prosocial interventions aim to promote voluntary actions that benefit others [1]. In particular, prosocial interventions for health encourage public participation in the promotion of health and well-being. Prosocial interventions can be associated with improved health outcomes, social well-being, and community connectedness [1–3]. Multi-disciplinary research suggests that promoting prosocial behaviors should be a public health priority [4]. *The Lancet* Commission on the COVID-19 pandemic [5] and the World Health Organization Commission for Social Connection [6] highlighted the need for greater prosocial behaviors [5].

A subset of prosocial interventions involves donations that are freely given for the benefit of others. Donation-based prosocial interventions for health encompass a wide range of types, including health services and products (e.g., diagnostics, vaccines), time, money, blood products, organs, data, and biospecimens. These interventions draw on behaviors that are rooted in generosity, reciprocity, and solidarity [7, 8]. Prosocial interventions leverage these behaviors to improve health and well-being in society. For example, a pay-it-forward approach to sexually treated infection (STI) prevention involves asking a person who has received a free STI test if they would like to donate an STI test or write a postcard spurring STI testing [1, 9]. Randomized controlled trials (RCTs) demonstrate that pay-it-forward interventions increase diagnostic test and vaccine uptake [1, 10].

Despite the growing use of donation-based prosocial interventions in health, there are important ethical issues that can be associated with them, which warrant deeper exploration. For instance, although prosocial interventions play a crucial role in facilitating life-saving organ transplantations and blood donations, there may be a risk of creating undue pressure among individuals who are asked to donate [11, 12]. Yet, the empirical ethics literature on donation-based prosocial interventions —beyond organ donations [12]— is limited. A prior systematic review documented the health outcomes of prosocial interventions [1] but did not evaluate non-health-related outcomes nor the associated ethical considerations. In addition, trials involving prosocial interventions have not comprehensively examined their associated ethical issues [9, 10]. This underscores the need to have a better empirical understanding of the actual benefits and ethical concerns associated with donation-based prosocial interventions. The purpose of this review is to fill this gap as well as identify strategies that have been used to mitigate ethical concerns or enhance the benefits associated with these interventions.

## Methods

We conducted a scoping review to map and synthesize the evidence on benefits and ethical concerns related to donation-based prosocial interventions, considering “benefits” to fall under the ethical principle of beneficence. We considered prosocial interventions as those promoting voluntary actions that help others, including donations of blood, organs, biospecimens, data, and money. We used a scoping review because it is well-suited to explore emerging areas of research or areas where there may be fragmented or emerging evidence [13], such as in the case of ethical issues of prosocial interventions. We followed the expanded Arksey and O’Malley framework for scoping reviews and used the PRISMA checklist for scoping reviews (PRISMA-ScR) [13–15]. We used the Arksey and O’Malley framework because it allowed for greater methodological flexibility and iterative refinement compared to other approaches. The review was not prospectively registered. Following the PRISMA checklist, we sought to answer the following research question: “What are the benefits and ethical concerns associated with donation-based prosocial interventions for health, and what mitigation strategies have been used or suggested in an effort to maximize the overall benefits of these interventions and minimize concerns?”

### Search strategy

We searched PubMed, PsycINFO and SCOPUS databases on July 1, 2025. We searched for empirical qualitative, quantitative, and mixed-methods studies of donation-based prosocial interventions in health published to this date. We focused on empirical literature, rather than the broader ethics literature on this topic. We also searched the NIH Reporter database and Clinical Trials repository for funded studies related to prosocial interventions. We used the following overarching search concepts in differing variations: “Prosocial” AND “Health” AND “Ethics.” Each category had a list of associated search terms, such as donation, public health, moral, and ethical (S1 Table). We also used citation tracking to find additional relevant studies for potential inclusion, including studies referenced in a previous systematic review of these interventions [1].

### Eligibility criteria and study screening

Our inclusion criteria were: (1) peer-reviewed studies of prosocial interventions involving a donation in a health context and (2) studies that specifically identified, explored or addressed ethical concerns related to these interventions. As mentioned earlier, we defined prosocial interventions as promoting voluntary actions that help others, including donations of blood, organs, biospecimens, data, and money. Only studies published in English were included. Two independent reviewers screened the titles and abstracts of publications using Covidence software. Studies that met the inclusion criteria were considered for full-text review. Review papers, opinion, and conceptual pieces were excluded. Conflicts were discussed and resolved by three study team members (TH, DS, and JT). Finally, the study characteristics of included studies were extracted into an Excel spreadsheet by each reviewer. This included information such as the author, date of publication, country, disease/donation area, study design, and relevant benefits and ethical issues.

### Data analysis and synthesis

We used descriptive statistics to describe the study characteristics. A codebook was developed to identify and extract relevant categories from the reported studies for the descriptive analysis (S2 Table). The initial codebook was inductively developed from a preliminary review of the studies, and then iteratively modified to meet our objective to identify benefits, ethical issues, and the strategies used to mitigate negative ethical issues. Each study was coded by two independent reviewers (TH, DS), and counts were generated for each code. Patterns of frequent codes were categorized with Excel software and used to identify cross-cutting issues across the studies. Frequency counts were reported, but were not interpreted as a proxy for ethical salience.

We identified four levels at which ethical concerns could occur in donation interventions, and consequently, where mitigation strategies could be deployed. These are the donor, recipient, provider, and system levels. We defined the donor as the initiator of the donation, and the recipient as the individual who receives the donation. The provider is the institution, program, or healthcare center that organizes the prosocial intervention and/or facilitates the donation between the donor and the recipient. The system refers to the larger environmental context in which the donation occurs, including the social, political, economic, and governmental context.

We assessed the quality of the included studies using the Quality Assessment for Diverse Studies (QuaDS) tool [16], and each study was quality-checked by two independent reviewers (TH, DS). The QuaDS tool is suited for assessing studies with heterogenous study designs and methods across a diverse range of fields. Consistent with other scoping reviews, quality ratings did not inform interpretation of descriptive findings.

## Results

### Search yield

**Fig. 1:**
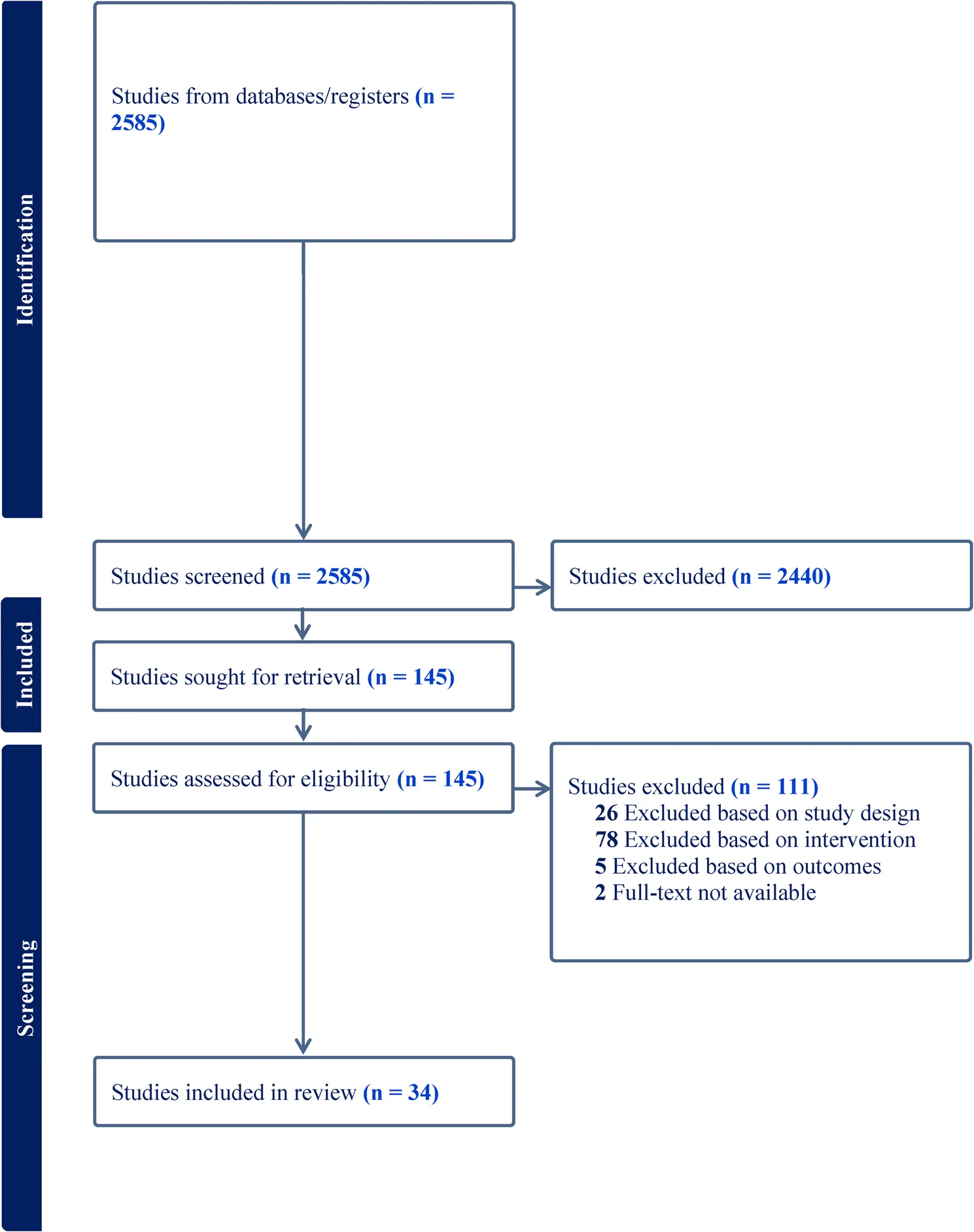
PRISMA flowchart of search results

The search identified 2,585 citations. A total of 145 studies were considered for full-text review (Fig. 1). After full-text review, 34 studies were included in this review [17–50].

### Study characteristics

There were 32 studies from high-income countries (HICs), and two from low-and-middle-income countries (LMICs). Thirty-one studies were observational, and three studies were experimental. Table 1 summarizes the characteristics of the included studies. Most studies were of moderate quality (S3 Table).

**Table 1:** Characteristics of included studies, *N* = 34

Characteristic	Number of studies (*n*, %)
Country	
High-income	32 (94%)
Low-and-middle-income	2 (6%)
Donated Item*	
Organ	14 (33%)
Biospecimens	11 (26%)
Data	6 (14%)
Money	4 (10%)
Blood	3 (7%)
Other material resources	4 (10%)
Study Design	
Observational	31 (91%)
Experimental	3 (9%)

*Some categories overlap with two types of donations involved in the intervention

The donation-based prosocial interventions we found involved donating organs (*n* = 14), biospecimens (*n* = 11), data (*n* = 6), money (*n* = 4), blood (*n* = 3), and other materials such as menstrual products and clothing (*n* = 4).

Next, we describe the results in the following categories: ethical principles, benefits, ethical concerns, and mitigation strategies.

### Ethical principles

Nearly all publications explicitly mentioned one or more of the following guiding ethical principles in the development or dissemination of the intervention: beneficence (*n* = 27), respect for autonomy (*n* = 23), non-maleficence (*n* = 16), transparency (*n* = 12), justice (*n* = 8), and solidarity (*n* = 5). However, we found that most studies did not empirically measure –or document—adverse events during or post-intervention.

### Benefits

Donation-based prosocial interventions resulted in morally desirable outcomes that included benefits in three domains: health (physical and psychological health), social, and economic. Social benefits included promoting generosity (*n* = 27), kindness (*n* = 19), community cohesiveness (*n* = 15), and trust (*n* = 12). We defined generosity as a trait marked by an attitude of sharing and giving, and kindness as showing concern and care for others. Economic benefits included promoting welfare (*n* = 6).

Several studies found that donation-based prosocial interventions improved physical health outcomes in the recipient, the donor, and the community (*n* = 23). For instance, organ transplantations were noted as life-saving interventions that reduced mortality in recipients [27, 37]. Other health-related benefits were generated through scientific discoveries and advancements that came from research on biospecimens or data donations [29], which may be considered secondary positive outcomes that may improve health in the future. For example, cancer patients voluntarily participated in research studies for altruistic reasons when there was no direct individual benefit to them, but in the hopes of improving cancer outcomes for future patients [35]. Additionally, several studies reported that donation interventions generated psychological or mental health benefits among donors (*n* = 10). For instance, one healthcare professional described the benefits of donating as receiving psychological benefits, such as the positive feeling one gets from contributing to the greater good [35].

There were several social benefits associated with donation-based interventions. These interventions provided opportunities for displaying generosity and giving to the community (*n* = 27) and opportunities to show kindness (*n* = 19). For instance, in one Danish study of individuals who donated placentas for research, participants viewed their prosocial acts as an opportunity to give back to the local health system that was free for all [34]. In another study exploring blood donors’ perspectives on extending their donations to biobanks for research, these contributions were regarded as an additional opportunity to help others, as participants viewed medical research as a means to promote societal well-being [22].

Trust also emerged as a common social benefit associated with donation-based prosocial interventions (*n* = 12). Many studies observed that donation interventions enhanced trust between donors, community members, organizations, and healthcare providers. One study on organ donation articulated the emergence of trust as a sequential process: increased information regarding the intervention led to transparency, which led to increased public trust, and ultimately increased people’s willingness to donate [24]. In a study of patients’ motivations for donating data and biospecimens to cancer research centers, participants stated that the opportunity to reciprocate the care that had been given to them was a motivator for making a donation themselves, thereby reinforcing trust in these institutions [18]. Additionally, participants’ motivation to donate was often due to the trust they had in clinicians and/or researchers to use their donations to help others [18].

Other social benefits of donation interventions included enhancing community cohesiveness (*n* = 15). For example, using community participatory research, an organization developed a list of acts of kindness for pregnant women that the public could participate in, such as buying necessities and offering instrumental support [38]. This generated a range of ways that the public could donate time, money and other things to support pregnant women, show kindness, and help decrease psychosocial stress [38]. These actions promoted community cohesion by mobilizing collective support for pregnant women and engaging community members in shaping positive social norms regarding how pregnant women are treated.

There were also economic benefits associated with donation-based prosocial interventions, such as improving welfare (*n* = 6). In one study, free menstrual products were provided to those in need [33]. Beyond saving money, receiving free menstrual hygiene products helped individuals who had been forced to self-isolate to re-engage in society. In turn, this allowed individuals to reduce school or work absences related to menstruation and thereby increase their productivity. This intervention also provided mental health benefits to recipients, such as feeling less alone, reducing stress, and feeling healthier [33].

### Ethical concerns

While donation-based prosocial interventions were associated with numerous benefits, we also found problematic ethical issues that may occur during their design and/or implementation. These issues were not formally assessed or measured in any of the included studies, but appeared as cross-cutting concerns in our analyses of them. The most common ethical concerns we identified were information related (*n* = 17), privacy violations (*n* = 14), undue pressure (*n* = 13), fairness (*n* = 11), and stigma (*n* = 10). These issues appeared at the donor, recipient, provider, and system levels.

A lack of understanding by potential donors, leading to the spread of inaccurate information, was a notable ethical concern with donation-based prosocial interventions (*n* = 17). For instance, in a qualitative study, some participants falsely believed that preventive HIV vaccine trials would benefit people who were already infected with HIV [19]. Another study noted that organ donations were often poorly understood by the public [32]. Specifically in public campaigns promoting organ donation, there were concerns of medical inaccuracies about what organ donations entail or overgeneralizations about potential benefits shared by campaigners in their effort to recruit more donors [27]. There were also concerns related to the lack of cultural or religious sensitivity in the communication of organ donor interventions, particularly for settings where organ donation may be restricted by cultural or religious beliefs. This left some communities feeling alienated by public campaigns that still included organ donation messages in such settings [32]. In other cases, participants misunderstood how research on biospecimen donations work. For instance, some donors expressed hopes of personally benefiting from the research even though no direct benefit was likely [35]. Overall, participating in donation-based interventions under misguided assumptions or with inaccurate information about the benefits or risks raises serious ethical concerns across the donor, recipient and provider levels.

Privacy violations also emerged in some studies (*n* = 14), affecting donors and/or recipients [26]. Concerns about privacy were often raised in qualitative research studies with participants and potential participants of donation interventions [18]. This concern was particularly pronounced when the donated item involved sensitive personal information, such as an individual’s HIV status [19, 21]. Donors, potential donors, and recipients sometimes preferred to keep their identity confidential, and participating in a donation intervention could inadvertently expose this information. Overall, donors and recipients, especially those from sexual and gender minorities, were particularly concerned about the unintentional disclosure of personal information during the donation process [21, 23].

Stigma was also mentioned as a concern for donors and recipients (*n* = 10), which often coincided with concerns about privacy described earlier. For instance, concerns about stigma were raised in a donation-based prosocial intervention that involved sharing electronic health records of children with intellectual and developmental disabilities for the advancement of health research [20]. Parents were concerned about the potential stigmatization of their children through the unintended disclosure of their child’s disability. Some studies (*n* = 13) noted the possibility of putting undue pressure on potential donors [24, 51]. For instance, there were concerns as to whether highly sensitized and emotional public appeals for donations would encroach on free and personal decision-making. This concern was common in prosocial interventions that involved blood or organ donations because the need and urgency associated with the donation could unintentionally induce feelings of pressure to donate [24]. Undue pressure was also commonly seen as a concern among more vulnerable populations [29]. Some participants in donation interventions could be more vulnerable to undue pressure based on their health condition, mental health, socioeconomic status, and/or level of education. There was also a concern of unduly creating a perceived moral burden on the donation recipient to reciprocate [31], which highlighted the ethical risks of linking receipt of health services with expectations of reciprocity when the recipient may not be able to do so.

The issue of fairness or lack thereof, especially in access to donated items, was also raised in many studies (*n* = 11). These concerns were expressed by participants of donation interventions who sought to know how their donations were being used and whether their distribution was fair. In addition, concerns about fairness emerged at the systems level, related to how fairly the benefits of donation interventions were distributed to the public, who made these decisions, and the rationale behind them [25, 52]. Finally, concerns about the equitable distribution of information about donation interventions was raised. For instance, an evaluation of public organ donation campaigns in Switzerland noted that if campaign messages are not properly translated into other languages, some minority groups might not benefit from these interventions due to the lack of awareness and information [25]. Concerns related to fairness and distributive justice were particularly salient during emergency settings, such as pandemics, when there was an acute need for scarce healthcare resources [52].

One study reported unintended consequences of donation-based interventions. Specifically, while evaluating the effect of giving and receiving social support during emergency situations such as an earthquake, there were a few reported instances of increased depression among givers of instrumental support (i.e., physical and financial aid), particularly among those who gave more support than they received [44]. However, this finding may have been confounded by the emergency situation itself and may be specific to disaster-affected settings. Another study noted that donation interventions may have the potential to inadvertently injure the self-esteem of charity donation recipients, particularly if the donor and recipient differ substantially in social status [49]. However, this risk is potentially attenuated by the feeling of gratitude and social connection fostered by communal generosity; that is, there is also evidence that gratitude may promote self-esteem [49]. Nonetheless, the potential for unintended adverse effects is an important ethical concern during the design and dissemination of donation-based prosocial interventions.

### Mitigation strategies

**Fig. 2:**
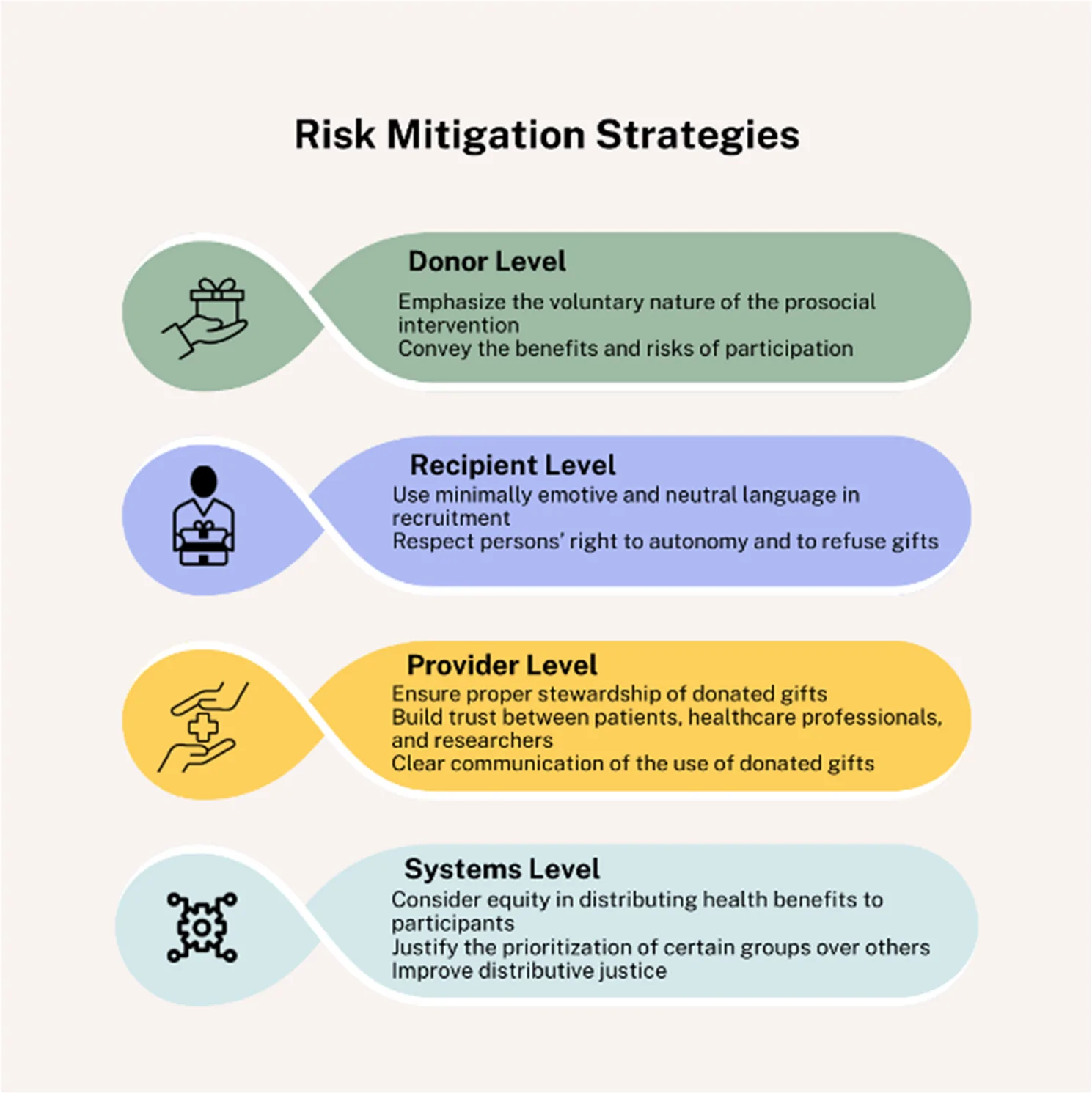
Mitigation strategies for donation-based prosocial interventions

The most common mitigation strategy to minimize or prevent ethical concerns related to donation-based prosocial interventions we identified was instituting a robust informed consent process for recipients and donors (*n* = 26) [18, 28, 45]. Others included the somewhat related need for clear messaging and communication (*n* = 24), establishing a trustworthy system and building trust (*n* = 18), protecting confidentiality (*n* = 14), ensuring appropriate stewardship of donations (*n* = 15), and incorporating religious/cultural sensitivity (*n* = 13). These mitigation strategies addressed ethical issues at the donor, recipient, provider and systems levels (Fig. 2).

In terms of consent, nearly all studies emphasized the importance of instituting a robust consent process at the donor level, providing all necessary information in a clear and accessible manner –which is in line with the standard practice in human subjects’ research. Notably, by adequately informing participants and using clear messaging, obtaining informed consent and combatting misinformation is simultaneously achieved [39]. Some studies suggested the use of dynamic or flexible consent, where the extent of the donation or involvement in the intervention can be changed after giving initial consent [20]. One study described the importance of informed consent broadly as making the donor aware of associated risks but also communicating the social value of the donation [22]. Clear, informative, and thoughtful messaging was believed to be important in mitigating concerns related to having inaccurate information or misunderstanding the intervention.

Several studies suggested mitigating unintended feelings of undue pressure for donors by making requests at an appropriate time and place [18, 34], emphasizing the “opt-in” or voluntary nature of the intervention, and clearly communicating the risk-benefit ratio of participating [18, 53]. Additionally, the literature highlights the importance of clearly conveying to donors that they will not face any repercussions should they choose not to participate [18, 37]. For recipients, minimizing undue pressure involves respecting the potential recipient’s autonomy to refuse a donation or not reciprocate one [31]. Another mitigation strategy is using minimally emotive or neutral language in recruiting the recipient to be a donor, which may help to minimize the risk of creating a moral burden or sense of obligation [24]. Relatedly, another study noted the importance of considering the ethical nature of the language used when communicating with recipients; for instance, the use of “gift” terminology may potentially downplay the magnitude of the sacrifice associated with the donation, particularly in the context of organ donations [50]. These considerations are also important and relevant during the creation of public campaigns and communication strategies.

Establishing a trustworthy system, particularly at the provider level, arose as an important mitigation strategy when developing and disseminating donation interventions in the community [19]. Notably, one study recommended building a strong institutional reputation to foster donor trust [22]. For instance, participants in a survey study on cancer biobanks expressed that they felt safe donating biospecimens because they trusted the health care professionals and researchers who would use them [18]. The authors identified trust as a facilitator of the intervention [18]. Another study emphasized that trust and transparency were integral when recruiting racially diverse groups to participate in prosocial interventions such as biospecimen donations [29].

Protecting the privacy and confidentiality of participants was another key mitigation strategy [28]. One study on promoting oral health through a web-based intervention described protecting the identity of participants by using digital means to shield identifying information from other participants. For example, organizing videoconference activities where attendees only see the program facilitator and enable anonymous chat windows [28]. Such considerations were also essential for maintaining trust and ensuring confidentiality once participants had been recruited for the intervention.

Transparency about the use of donation was also crucial. For instance, one study investigated whether blood donors and non-donors found it acceptable to conduct routine genomic testing on their donated blood. The authors found that there was some support for certain limited kinds of testing (e.g., to identify rare blood types), but partial to little support for more extensive testing [30]. Focus group participants in a study on data and biospecimen donation mentioned that a lack of transparency on the use of donated data would damage trust, and more transparency would build trust and make them more likely to share their data [20]. For data donations for research, transparency on how these data would be used was paramount, particularly, donors were open to sharing data for beneficial scientific projects [22]. On the other hand, some participants expressed mistrust of the healthcare system and collected data for research due to historical examples of unethical research practices on minority groups [23]. Other studies cited medical mistrust in organ donations [45], and among diverse communities for biospecimen donations [29]. These findings illustrated the importance of building trustworthy systems and fostering institutional trust in research and healthcare systems [17–19, 22].

Ensuring good stewardship of gifts and transparency in their distribution was also essential. Several studies highlighted that decision-makers and policymakers ought to consider equity in the distribution of benefits at the systems level [23, 25]. If it is necessary to prioritize certain groups due to limited resources, the selection process for recipients should be clearly communicated and justified, particularly concerning the criteria used to determine eligibility. Appropriate governance and oversight, transparent decision-making, and clear justifications are crucial strategies for mitigating ethical concerns at the systems level. Moreover, an understanding of the structural injustices at play is crucial for identifying where and how distributive justice can be promoted [25]. Solidarity was referenced as a powerful ethical principle to embody in making these decisions at the systems level [8].

Finally, incorporating religious and cultural sensitivity was highlighted as a valuable mitigation strategy to address the concerns of religious and cultural groups, particularly in tissue donations [45]. This mitigation strategy could be incorporated at all levels to address ethical concerns involving members of minority groups. In one study, community engagement and transparency about biospecimen donation were leveraged to build public trust among ethnic and racial minorities [29]. In particular, community outreach was conducted to provide biospecimen education in both English and the native languages of the potential participants, illustrating an example of culturally responsive recruitment strategies in donation-based interventions.

## Discussion

Interventions aimed at promoting kindness, solidarity, and generosity—such as donation-based prosocial interventions—are often considered inherently beneficial and socially valuable in society. However, there can be ethical issues associated with them. This scoping review synthesized published evidence on donation-based prosocial interventions, providing a comprehensive description of the associated ethical issues. We found that donation-based interventions can confer several health, social, and economic benefits. However, they may involve a range of ethical concerns that can be anticipated and mitigated. Our findings are relevant for future studies and interventions that leverage a prosocial approach in their design and implementation.

Our review suggests that generosity and giving behavior is one of the most prominent benefits of donation-based interventions. In about 80% of the studies we reviewed, people who participated in prosocial interventions were inclined to give generously to others; donation interventions provide an opportunity to leverage the human instinct to help others by giving [4, 54]. In addition, helping others through giving can promote physical [10] and mental health [3] benefits in the recipient and donor. For instance, recipients of donation interventions reported improved mental health benefits, such as feeling less alone [33]. This aligns with recommendations for increased social connectedness from the WHO Commission on Social Connection [6].

Our findings suggest that prosocial behavior can also increase social cohesiveness in a community [44, 55]. One study discussed the crucial role of reciprocity (i.e., giving and receiving) in strengthening communal relationships, and that reciprocal exchanges are essential for enhancing interconnectedness and social cohesion [44]. Reciprocity is increasingly noted as crucial in health intervention research. Several studies have demonstrated that reciprocity-based donation strategies increase diagnostic test uptake and vaccine uptake [1, 52]. Given the disparities that exist within populations in health and wealth, reciprocity can also be viewed as a means of promoting distributive justice through redistribution of resources [55]. Further, donation interventions provide an opportunity for the public to actively engage in the promotion of health and well-being, which can enhance community cohesion.

Nevertheless, there can be important ethical concerns associated with these interventions. A common issue relates to the spread of inaccurate information or misunderstandings about donation interventions, which can negatively affect achieving their goals. For instance, inaccurate information about the benefits and risks of tissue donations can limit the benefits of these interventions, either through limited or misinformed participation leading to unintended consequences [11, 12]. The risk of inaccurate information can be mitigated by carefully creating public awareness campaigns that communicate the goals of the intervention, ideally in collaboration with community members themselves [19, 43]. Additionally, ethical concerns related to potential undue pressure and privacy violations should be given serious consideration throughout every stage of intervention design and implementation, to ensure the protection of potential participants and overall public trust.

### Implications

There are several implications of this work. First, public trust in the medical and health system seems to be an important facilitator of donation-based prosocial interventions [17, 18]. However, ensuring that these systems are trustworthy at the outset is paramount. Our review adds to other literature on the importance of creating trustworthy healthcare systems to earn public trust and sustaining that trust to improve health interventions. For instance, some minoritized groups avoid donating biospecimens because of historical unethical practices, giving the example of the ‘Tuskegee Syphilis Study’ [23]. As a mitigation strategy, donor trust can be rebuilt and strengthened through informed consent, transparency, and appropriate stewardship of donations that are freely given for research [19, 29].

Of note, with the exception of some organ and blood donation studies, we found that ethical issues do not seem to be systematically assessed in intervention research [9, 56, 57]. However, it is crucial to consider ethical issues in other public health interventions that involve giving, as failure to anticipate and address them may lead to unintended adverse consequences.

### Strengths and limitations

As donation-based prosocial interventions become more common and broadly implemented in public health, it is essential to evaluate how they might unintentionally cause harm and take measures to minimize that possibility. A strength of our review is that it considered a broad range of evidence, to synthesize both the range of benefits and negative ethical issues that may arise in the development and dissemination of donation interventions. Additionally, we synthesized the findings into four levels at which ethical issues could occur, which can help public health researchers and policymakers who seek to use donation-based interventions to consider important ethical issues. Specifically, the four levels–donor, recipient, provider, and system– may be used as a guide for developing mitigation strategies at each level to ensure that the benefits of donation interventions are maximized, and ethical concerns are minimized.

Despite the importance of our findings, this review has some limitations. First, the scoping review methodology prioritized mapping a broad range of evidence across the fields of medicine, healthcare, and public health. There may have been prosocial interventions that involved donations that were not captured by our search strategy because they did not use these specific terms. At the same time, our search identified diverse categories of donation-based prosocial interventions (e.g., organs, biospecimens, money), making it likely that the key ethical issues were still identified by our search. Some reports of the ethical issues associated with donation-based prosocial interventions may not have used our search terms in the title or abstract and would therefore not be included here. Second, as only English-language studies were included, there may have been key literature in other languages that was missed, which is a limitation of this review. Third, there was variation in how donation interventions were defined and measured, which may have limited our evaluation of these studies. Additionally, some concepts of the constructs related to donation interventions, such as reciprocity and gratitude, do not have validated measures. However, several codes included in our descriptive analysis of ethical issues were common among many of the included studies, which suggests the potential validity of our approach to identifying key constructs. Finally, we did not focus on conceptual ethics scholarship and similar reports, which may have included additional relevant empirical findings. Overall, these limitations indicate opportunities for further research, especially in the measurement of risks and prospective trials.

## Conclusion

Our review highlights the need to explicitly consider the ethical issues of donation-based prosocial interventions in their design and dissemination. By ensuring informed consent, transparency, confidentiality, and donor trust, donation interventions may promote benefits in terms of health and generosity while minimizing ethical concerns related to them. Future research should prospectively examine the ethical concerns identified in this review along with strategies to mitigate them.

## Supplementary Information

Supplementary Material 1. S1 Table. Search strategy. S2 Table. Codebook. S3 Table. QuaDS Assessment.

## Data Availability

The findings of this review were drawn from published articles, which can be retrieved on public databases using the search string provided in the Supplementary Materials file.

## Abbreviations

QuaDS: Quality Assessment for Diverse Studies
RCT: Randomized controlled trial
SESH: Social engagement to spur health
STI: Sexually transmitted infection
WHO: World Health Organization

## References

[1] Byrne M, Tan RKJ, Wu D, Marley G, Hlatshwako TG, Tao Y, et al. Prosocial Interventions and Health Outcomes: A Systematic Review and Meta-Analysis. JAMA Netw Open. 2023;6(12):e2346789. 10.1001/jamanetworkopen.2023.46789. PMC10709779 38064214

[2] Ibanez A, Matallana D, Miller B. Can prosocial values improve brain health? Front Neurol. 2023;14. 10.3389/fneur.2023.1202173. PMC10278355 37342774

[3] Varma MM, Chen D, Lin X, Aknin LB, Hu X. Prosocial behavior promotes positive emotion during the COVID-19 pandemic. Emotion. 2023;23(2):538–53. 10.1037/emo0001077. 35298223

[4] Kubzansky LD, Epel ES, Davidson RJ. Prosociality should be a public health priority. Nat Hum Behav. 2023;7(12):2051–3. 10.1038/s41562-023-01717-3. PMC10840689 37857873

[5] Sachs JD, Karim SSA, Aknin L, Allen J, Brosbøl K, Colombo F, et al. The Lancet Commission on lessons for the future from the COVID-19 pandemic. Lancet. 2022;400(10359):1224–80. 10.1016/s0140-6736(22)01585-9. PMC9539542 36115368

[6] WHO. WHO Commission on Social Connection. 2024. https://www.who.int/groups/commission-on-social-connection.

[7] Wathuta J. A Look at Uganda’s Early HIV Prevention Strategies Through a Moderate ‘African’ Communitarian Lens. Dev World Bioeth. 2018;18(2):109–18. 10.1111/dewb.12139. 28032470

[8] Gould CC. Solidarity and the problem of structural injustice in healthcare. Bioethics. 2018;32(9):541–52. 10.1111/bioe.12474. 30044895

[9] Yang F, Zhang TP, Tang W, Ong JJ, Alexander M, Forastiere L, et al. Pay-it-forward gonorrhoea and chlamydia testing among men who have sex with men in China: a randomised controlled trial. Lancet Infect Dis. 2020;20(8):976–82. 10.1016/S1473-3099(20)30172-9. PMC8957706 32530426

[10] Li KT, Tang W, Wu D, Huang W, Wu F, Lee A, et al. Pay-it-forward strategy to enhance uptake of dual gonorrhea and chlamydia testing among men who have sex with men in China: a pragmatic, quasi-experimental study. Lancet Infect Dis. 2019;19(1):76–82. 10.1016/S1473-3099(18)30556-5. PMC6347395 30587296

[11] Ullah K, Dogar AW, Ochani S, Ahmad HB, Hasibuzzaman MA. Donor’s ability to comprehend and carefully assess the benefits and risks of donation in living donor liver transplantation: a Pakistani perspective – a short communication. Annals Med Surg. 2023;85(9):4646–8. 10.1097/ms9.0000000000001121. PMC10473353 37663723

[12] Arai N, Takimoto Y, Nakazawa E, Akabayashi A. Considerations on the Factors Influencing Living Kidney Donors’ Autonomous Decision-Making: A Systematic Literature Review. Transplantation Proceedings. 2018;50(10):3036–44. 10.1016/j.transproceed.2018.06.015. 30577163

[13] Arksey H, O’Malley L. Scoping studies: towards a methodological framework. Int J Soc Res Methodol. 2005;8(1):19–32. 10.1080/1364557032000119616.

[14] Levac D, Colquhoun H, O’Brien KK. Scoping studies: advancing the methodology. Implement Sci. 2010;5(1):69. 10.1186/1748-5908-5-69. PMC2954944 20854677

[15] Tricco AC, Lillie E, Zarin W, O’Brien KK, Colquhoun H, Levac D, et al. PRISMA Extension for Scoping Reviews (PRISMA-ScR): Checklist and Explanation. Ann Intern Med. 2018;169(7):467–73. 10.7326/M18-0850. 30178033

[16] Harrison R, Jones B, Gardner P, Lawton R. Quality assessment with diverse studies (QuADS): an appraisal tool for methodological and reporting quality in systematic reviews of mixed- or multi-method studies. BMC Health Serv Res. 2021;21(1):144. 10.1186/s12913-021-06122-y. PMC7885606 33588842

[17] Nguyen AQ, Anjum SK, Halpern SE, Kumar K, Van Pilsum Rasmussen SE, Doby B, et al. Willingness to Donate Organs Among People Living With HIV. JAIDS J Acquir Immune Defic Syndr. 2018;79(1):e30–6. 10.1097/qai.0000000000001760. PMC6092203 29781880

[18] Yip S, Fleming J, Shepherd HL, Walczak A, Clark J, Butow P. As Long as You Ask: A Qualitative Study of Biobanking Consent—Oncology Patients’ and Health Care Professionals’ Attitudes, Motivations, and Experiences—the B-PPAE Study. Oncologist. 2018;24(6):844–56. 10.1634/theoncologist.2018-0233. PMC6656517 30413662

[19] Toledo L, McLellan-Lemal E, Arreola S, Campbell C, Sutton M. African-American and Hispanic Perceptions of HIV Vaccine Clinical Research: A Qualitative Study. Am J Health Promotion. 2014;29(2):e82–90. 10.4278/ajhp.130125-QUAL-48. 24432823

[20] Andrews SM, Raspa M, Edwards A, Moultrie R, Turner-Brown L, Wagner L, et al. Just tell me what’s going on: The views of parents of children with genetic conditions regarding the research use of their child’s electronic health record. J Am Med Inform Assoc. 2020;27(3):429–36. 10.1093/jamia/ocz208. PMC7025353 31913479

[21] Lessard D, Dubé K, Bilodeau M, Keeler P, Margolese S, Rosenes R, et al. Willingness of Older Canadians with HIV to Participate in HIV Cure Research Near and After the End of Life: A Mixed-Method Study. AIDS Res Hum Retroviruses. 2022;38(8):670–82. 10.1089/aid.2022.0006. PMC9483839 35778845

[22] Raivola V, Snell K, Helén I, Partanen J. Attitudes of blood donors to their sample and data donation for biobanking. Eur J Hum Genet. 2019;27(11):1659–67. 10.1038/s41431-019-0434-1. PMC6871534 31147625

[23] Cicero EC, Lunn MR, Obedin-Maliver J, Sunder G, Lubensky ME, Capriotti MR et al. Acceptability of Biospecimen Collection Among Sexual and/or Gender Minority Adults in the United States. sgrlgbtq. (4):311–44. 10.1891/LGBTQ-2022-0021. PMC11374103 39234441

[24] Hansen SL, Pfaller L, Schicktanz S. Critical analysis of communication strategies in public health promotion: An empirical-ethical study on organ donation in Germany. Bioethics. 2021;35(2):161–72. 10.1111/bioe.12774. 32557682

[25] Merminod G, Benaroyo L. Ethical issues in public health communication: Practical suggestions from a qualitative study on campaigns about organ donation in Switzerland. Patient Educ Couns. 2022;105(4):881–6. 10.1016/j.pec.2021.07.012. 34281724

[26] Snyder J, Crooks VA. Is there room for privacy in medical crowdfunding? J Med Ethics. 2021;47(12):e49–e. 10.1136/medethics-2020-106676. 33208481

[27] Waterman AD, Wood EH, Ranasinghe ON, Faye Lipsey A, Anderson C, Balliet W, et al. A Digital Library for Increasing Awareness About Living Donor Kidney Transplants: Formative Study. JMIR Form Res. 2020;4(7):e17441. 10.2196/17441. PMC7404010 32480362

[28] Carpenter SM, Greer ZM, Newman R, Murphy SA, Shetty V, Nahum-Shani I. Developing Message Strategies to Engage Racial and Ethnic Minority Groups in Digital Oral Self-Care Interventions: Participatory Co-Design Approach. JMIR Form Res. 2023;7:e49179. 10.2196/49179. PMC10750234 38079204

[29] Dang JHT, Chen MS Jr. Time, trust, and transparency: Lessons learned from collecting blood biospecimens for cancer research from the Asian American community. Cancer. 2018;124(S7):1614–21. 10.1002/cncr.31168. PMC5894865 29578601

[30] Thorpe R, Jensen K, Masser B, Raivola V, Kakkos A, von Wielligh K, et al. Donor and non-donor perspectives on receiving information from routine genomic testing of donor blood. Transfusion. 2023;63(2):331–8. 10.1111/trf.17215. PMC10107456 36478364

[31] Nakazawa E, Shaw MH, Akabayashi A. The Ethical Acceptability of a Recipient’s Choice of Donor in Directed and Nondirected Transplantation: Japanese Perspective. Camb Q Healthc Ethics. 2023;32(2):216–21. 10.1017/S0963180122000214. 36330827

[32] Faherty G, Williams L, Noyes J, Mc Laughlin L, Bostock J, Mays N. Analysis of content and online public responses to media articles that raise awareness of the opt-out system of consent to organ donation in England. Front Public Health. 2022;10. 10.3389/fpubh.2022.1067635. PMC9751921 36530724

[33] Massengale KE, Bowman KM, Comer LH, Van Ness S. Breaking the period product insecurity cycle: An observational study of outcomes experienced by recipients of free period products in the United States. Women’s Health. 2024;20:17455057241267104. 10.1177/17455057241267104. PMC11273700 39051651

[34] Lind U, Mose T, Knudsen LE. Participation in environmental health research by placenta donation - a perception study. Environ Health. 2007;6:36. 10.1186/1476-069x-6-36. PMC2211473 18034882

[35] Kleiderman E, Avard D, Black L, Diaz Z, Rousseau C, Knoppers BM. Recruiting Terminally Ill Patients into Non-Therapeutic Oncology Studies: views of Health Professionals. BMC Med Ethics. 2012;13(1):33. 10.1186/1472-6939-13-33. PMC3529687 23216847

[36] McIntyre J, Pratt C, Pentz RD, Haura EB, Quinn GP. Stakeholder perceptions of thoracic rapid tissue donation: An exploratory study. Soc Sci Med. 2013;99:35–41. 10.1016/j.socscimed.2013.08.035. PMC3869974 24355468

[37] Jernigan M, Fahrenwald N, Harris R, Tsosie U, Baker LO, Buchwald D. Knowledge, beliefs, and behaviors regarding organ and tissue donation in selected tribal college communities. J Community Health. 2013;38(4):734–40. 10.1007/s10900-013-9672-2. PMC3706512 23504267

[38] Jones L, Lu MC, Lucas-Wright A, Dillon-Brown N, Broussard M, Wright K, et al. One Hundred Intentional Acts of Kindness toward a Pregnant Woman: building reproductive social capital in Los Angeles. Ethn Dis. 2010;20(1 Suppl 2):S2–36. PMC3787313 20629245

[39] Baird W, Jackson R, Ford H, Evangelou N, Busby M, Bull P, et al. Holding personal information in a disease-specific register: the perspectives of people with multiple sclerosis and professionals on consent and access. J Med Ethics. 2009;35(2):92–6. 10.1136/jme.2008.025304. 19181880

[40] Harkins KA, Kullgren JT, Bellamy SL, Karlawish J, Glanz K. A Trial of Financial and Social Incentives to Increase Older Adults’ Walking. Am J Prev Med. 2017;52(5):e123–30. 10.1016/j.amepre.2016.11.011. 28062271

[41] Hobson O, Lumsdaine J, Sherif A, Oniscu GC. A Home Education Service to Increase Knowledge of Treatment Options and Improve Attitudes to Living Donor Kidney Transplantation. Prog Transpl. 2023;33(1):95–9. 10.1177/15269248221145041. 36562149

[42] Gordon EJ, Romo E, Amórtegui D, Rodas A, Anderson N, Uriarte J, et al. Implementing culturally competent transplant care and implications for reducing health disparities: A prospective qualitative study. Health Expect. 2020;23(6):1450–65. 10.1111/hex.13124. PMC7752187 33037746

[43] Dubé K, Villa TJ, Taylor J, Kaytes A, Moore DJ, Little SJ, et al. A Community-Driven Framework to Prioritize the Use of Donated Human Biological Materials in the Context of HIV Cure-Related Research at the End of Life. Pathog Immun. 2023;8(1):1–16. 10.20411/pai.v8i1.583. PMC10225111 37252012

[44] Pandit A, Nakagawa Y. How Does Reciprocal Exchange of Social Support Alleviate Individuals’ Depression in an Earthquake-Damaged Community? Int J Environ Res Public Health. 2021;18(4). 10.3390/ijerph18041585. PMC7914432 33567495

[45] Clark NL, Coe D, Newell N, Jones MNA, Robb M, Reaich D, et al. I am in favour of organ donation, but I feel you should opt-in—qualitative analysis of the #options 2020 survey free-text responses from NHS staff toward opt-out organ donation legislation in England. BMC Med Ethics. 2024;25(1):47. 10.1186/s12910-024-01048-6. PMC11031982 38643137

[46] Papadimos TJ, Papadimos AT. The student and the ovum: The lack of autonomy and informed consent in trading genes for tuition. Reproductive Biology Endocrinol. 2004;2(1):56. 10.1186/1477-7827-2-56. PMC479702 15248892

[47] Boulware LE, Hill-Briggs F, Kraus ES, Melancon JK, Senga M, Evans KE, et al. Identifying and addressing barriers to African American and non-African American families’ discussions about preemptive living related kidney transplantation. Prog Transpl. 2011;21(2):97–104. 10.1177/152692481102100203. quiz 5. 21736237

[48] Philpot SJ, Aranha S, Pilcher DV, Bailey M, Randomised D, Blind. Controlled Trial of the Provision of Information about the Benefits of Organ Donation during a Family Donation Conversation. PLoS ONE. 2016;11(6):e0155778. 10.1371/journal.pone.0155778. PMC4913899 27322832

[49] Hu CS, Zhang H, Munroe M, Huang C, Cao Y, Yan J, et al. Peer charity donation, gratitude, and self-esteem among left-behind children. Br J Soc Psychol. 2023;62(2):725–42. 10.1111/bjso.12590. 36266767

[50] Shaw R. Perceptions of the gift relationship in organ and tissue donation: Views of intensivists and donor and recipient coordinators. Soc Sci Med. 2010;70(4):609–15. 10.1016/j.socscimed.2009.10.062. 19931963

[51] Hansen SL, Eisner MI, Pfaller L, Schicktanz S. Are You In or Are You Out?! Moral Appeals to the Public in Organ Donation Poster Campaigns: A Multimodal and Ethical Analysis. Health Commun. 2018;33(8):1020–34. 10.1080/10410236.2017.1331187. 28622010

[52] O’Sullivan L, Aldasoro E, O’Brien Á, Nolan M, McGovern C, Carroll Á. Ethical values and principles to guide the fair allocation of resources in response to a pandemic: a rapid systematic review. BMC Med Ethics. 2022;23(1):70. 10.1186/s12910-022-00806-8. PMC9261249 35799187

[53] Filler G, de Ferris MED-G, Elliott L. Marginal parent donors—Process and ethics. Pediatr Transplant. 2021;25(5):e14062. 10.1111/petr.14062. 34076958

[54] Haller E, Lubenko J, Presti G, Squatrito V, Constantinou M, Nicolaou C, et al. To Help or Not to Help? Prosocial Behavior, Its Association With Well-Being, and Predictors of Prosocial Behavior During the Coronavirus Disease Pandemic. Front Psychol. 2021;12:775032. 10.3389/fpsyg.2021.775032. PMC8877810 35222147

[55] Meier S. A survey of economic theories and field evidence on pro-social behavior. 2006.

[56] Galárraga O, Bohlen LC, Dunsiger SI, Lee HH, Emerson JA, Boyle HK, et al. Small sustainable monetary donation-based incentives to promote physical activity: A randomized controlled trial. Health Psychol. 2020;39(4):265–8. 10.1037/hea0000818. PMC7078055 31750676

[57] Haydon MD, WL C, Danny MFM, Sonja R L, and, Bower JE. Kindness interventions for early-stage breast cancer survivors: An online, pilot randomized controlled trial. J Posit Psychol. 2023;18(5):743–54. 10.1080/17439760.2022.2093786.

