# Supplementary material for: Ethical issues in donation-based prosocial interventions for health: a scoping review
Source: BMC Med Ethics. 2026 Jul 22;27:223. doi: 10.1186/s12910-026-01559-4 (PMC13711855; doi:10.1186/s12910-026-01559-4)
Supplement: Supplementary file 1 [file 12910_2026_1559_MOESM1_ESM.docx]

**Ethical Issues in Donation-Based Prosocial Interventions for Health: A Scoping Review**

**Supporting Information**

**S1 Table. Search strategy**

| PubMed Search String | (("Prosocial"[tiab] OR "prosociality"[tiab] OR "pay-it-forward"[tiab] OR "pay it forward"[tiab] OR "donation"[tiab] OR "generosity"[tiab] OR "reciprocity"[tiab] OR "Gift-giving"[tiab] OR "Altruism"[tiab] OR "kindness"[tiab] OR "charity"[tiab] OR "cash gifts"[tiab] OR "gift"[tiab] OR "donat*"[tiab] OR "acts of kindness"[tiab] OR "helping behavior"[tiab] OR "altruistic"[tiab] OR "gratitude"[tiab]) AND ("Health"[tiab] OR "Health intervention*"[tiab] OR "Public Health"[tiab] OR "Health promotion"[tiab] OR "Health behavior"[tiab] OR "community engagement"[tiab] OR "community health"[tiab])) AND ("Ethics"[tiab] OR "Ethical Issue*"[tiab] OR "Ethical concern*"[tiab] OR "Moral*"[tiab] OR "Coerci*"[tiab] OR "Trust"[tiab] OR "Public health ethics"[tiab] OR "common good"[tiab] OR "Justice"[tiab] OR "Equity"[tiab]) |
| --- | --- |

**S2 Table. Codebook**

1. **Region (World bank regions)**

1.1. Low income

1.2. Middle income

1.3. High income

2. **Type of prosocial intervention**

2.1. Reciprocity-based

2.2. Non-reciprocity based

3. **Donated item**

3.1. Money

3.2. Time

3.3. Medical:

3.3.1.1. Blood

3.3.1.2. Data

3.3.1.3. Biospecimen/other tissue

3.3.1.4. Organ

3.4. Material resources

4. **Fundamental ethical principles cited**

4.1. Respect for autonomy

4.2. Beneficence

4.3. Non-maleficence

4.4. Justice

4.5. Transparency

4.6. Solidarity

5. **Positive ethical issues/benefits of intervention**

5.1. Physical health benefits

5.2. Psychological/Mental health benefits/

5.3. Social benefits

5.3.1. Increased community cohesion

5.3.2. Decreasing stigma

5.3.3. Building trust

5.3.4. Kindness

5.3.5. Generosity/Giving behavior

5.3.6. Reciprocity

5.4. Economic benefits

5.4.1. Improving welfare
5.4.2. Employment

6. **Negative ethical issues/concerns discussed or described**

6.1. Undue pressure

6.2. Stigma

6.3. Privacy

6.4. Fairness

6.5. Inaccurate Information/Misinformation

7. **Ethics Mitigation strategies**

7.1. Informed consent

7.2. Stewardship of gifts

7.3. Establishing a trustworthy system

7.4. Confidentiality

7.5. Opportunities for feedback

7.6. Re-consent

7.7. Clear messaging

7.8. Cultural/religious sensitivity

8. **Study design**

8.1. Observational

8.2. Experimental

**S3 Table. QuaDS Assessment**

| **Quality assessment (score from 0-3)** | | | | | |
| --- | --- | --- | --- | --- | --- |
| **Author (first)** | **Concepts related to prosociality** | **Study design appropriateness** | **Description of setting and target population** | **Stakeholder involvement** | **Limitations addressed** |
| Nguyen, Anh Q. | 3 | 2 | 2 | 3 | 3 |
| Yip, Sonia | 3 | 2 | 2 | 3 | 2 |
| Toledo, Lauren | 3 | 2 | 2 | 3 | 3 |
| Andrews, Sara | 3 | 2 | 0 | 3 | 3 |
| Lessard, David | 3 | 2 | 2 | 3 | 3 |
| Raivola, Vera | 3 | 2 | 2 | 2 | 1 |
| Cicero, Ethan | 3 | 2 | 0 | 0 | 3 |
| Hansen, Solveig Lena | 3 | 2 | 0 | 3 | 3 |
| Clark, Natalie L. | 3 | 2 | 0 | 3 | 2 |
| Merminod, Gilles | 3 | 2 | 0 | 1 | 1 |
| Snyder, Jeremy | 3 | 2 | 1 | 2 | 1 |
| Waterman, Amy | 3 | 2 | 1 | 2 | 2 |
| Philpot, Steve John | 3 | 3 | 3 | 2 | 3 |
| Pandit, Arpana | 3 | 2 | 2 | 2 | 2 |
| Carpenter, Stephanie | 3 | 2 | 1 | 3 | 3 |
| Dang, Julie | 3 | 2 | 1 | 3 | 3 |
| Hobson, Orla | 3 | 2 | 2 | 3 | 3 |
| Gordon, Elisa | 3 | 2 | 0 | 3 | 2 |
| Karine Dube | 3 | 2 | 0 | 3 | 1 |
| Thorpe, Rachel | 2 | 2 | 1 | 2 | 3 |
| Nakazawa, Eisuke | 3 | 2 | 0 | 0 | 3 |
| O'Sullivan, Lydia | 3 | 2 | 0 | 3 | 3 |
| Faherty, Georgia | 3 | 2 | 0 | 3 | 3 |
| Massengale, Kelley | 3 | 2 | 2 | 2 | 3 |
| Lind, Uffe | 2 | 2 | 0 | 1 | 1 |
| Papadimos, Thomas | 3 | 2 | 0 | 0 | 3 |
| Kleiderman, Erika | 3 | 2 | 0 | 3 | 3 |
| McIntyre, Jessica | 3 | 2 | 0 | 3 | 3 |
| Boulware, L. Ebony | 3 | 2 | 2 | 3 | 3 |
| Jernigan, Meghan | 3 | 2 | 0 | 3 | 3 |
| Jones, Loretta | 3 | 2 | 0 | 3 | 1 |
| Baird, W | 3 | 2 | 0 | 1 | 1 |
| Harkins Kristin | 3 | 3 | 0 | 0 | 2 |
| Hu, Chao | 3 | 3 | 0 | 3 | 2 |
| Shaw, Rhonda | 3 | 2 | 0 | 0 | 2 |
| A score of 0 indicates that the assessment criterion has not been met. A score 3 indicates that the assessment criterion has been fully met. A score of 1-2 indicates that the criterion has been partially met. | | | | | |
